# Mapping the Relationship Between Diffusion Characteristics of Warm-Mix Recycled Asphalt on Molecular Dynamics (MD) and High-Low Temperature Properties of Mixtures

**DOI:** 10.3390/ma18122740

**Published:** 2025-06-11

**Authors:** Xin Jin, Shanshan Meng, Haoxuan Fu, Qi Zhao, Deli Li, Zhuolin Li, Ye Yang, Yanhai Yang, Jiupeng Zhang, Qingyue Zhou

**Affiliations:** 1 School of Transportation and Geomatics Engineering, Shenyang Jianzhu University, Shenyang 110168, China; mss202505@163.com (S.M.); yqbx35489@163.com (H.F.); lideli831@163.com (D.L.); yangye@sjzu.edu.cn (Y.Y.); yangyanhai168@126.com (Y.Y.); 2 School of Highway, Chang’an University, Xi’an 710064, China; zhjiupeng@chd.edu.cn; 3 Jiangsu Zengguang New Material Technology Co., Ltd., Nantong 226602, China; jszgkjgfyxgs@126.com; 4 School of Management, Shenyang Jianzhu University, Shenyang 110168, China; dbdxzq@163.com; 5 Liaoning Transportation Research Institute Co., Ltd., Shenyang 110015, China; 15040026702@163.com

**Keywords:** warm-mix recycling, MD, high-low temperature properties of mixtures, diffusion characteristics

## Abstract

Warm-mix recycled asphalt (WMA-R) technology for reclaimed asphalt pavement (RAP) significantly reduces energy consumption and environmental pollution while maintaining the performance of asphalt mixtures. Significant progress has been made at home and abroad in evaluating the impact of regenerated asphalt mixtures on the performance of regenerated asphalt. However, the performance improvement of WMA-R depends on the effective diffusion of regenerated agents and their interaction mechanism with aged asphalt, which has not been fully studied. This paper systematically studies the diffusion characteristics of biomimetic-based warm-mix regenerant in aged asphalt and its impact on the high- and low-temperature performance of asphalt mixtures through MD and experimental verification. The results show that biomimetic-based warm-mix regenerant can significantly improve the diffusion performance of aged asphalt. Through the rutting test and low-temperature bending test, the significant improvement of the biomimetic-based warm-mix regenerant in the rutting resistance and crack resistance of asphalt mixtures was verified.

## 1. Introduction

With the rapid development of road transportation infrastructure, the recycling of asphalt pavement has become crucial for sustainable development. Although conventional hot recycling techniques can effectively reclaim aged asphalt mixtures, the high-temperature construction process not only consumes substantial energy but also emits harmful gases, causing adverse environmental impacts. In the 1990s, Europe took the lead in proposing warm-mix technology [1] to reduce greenhouse gas emissions. In 2007, Rajib B. Mallick [2] and others combined warm-mix technology with recycling technology and carried out a large number of tests, thus triggering a research upsurge in warm-mix recycling technology. Significant progress has been made domestically and internationally in evaluating the influence of regenerants on the performance of recycled asphalt mixtures. Current research has gradually shifted toward in-depth investigations of regeneration mechanisms, along with active exploration of regenerant selection and formulation technologies [3]. WMA-R significantly reduces energy consumption and environmental pollution by lowering construction temperatures while maintaining asphalt mixture performance. However, the performance enhancement of WMA-R relies heavily on the effective diffusion of regenerants and their interaction mechanisms with the aged asphalt-a process that remains insufficiently studied.

Previous analyses have mainly focused on the macroscopic and microscopic levels, making it difficult to explain phenomena at the molecular scale. At present, molecular dynamics simulation (MD) has been applied to relevant research on asphalt. Based on the principles of classical mechanics, MD solves the numerical solutions of Newton’s equations of motion for all atomic nuclei in the system through the total potential energy of the molecular system, analyzes the evolution of the positions and velocities of atomic nuclei over time, and calculates the dynamic and thermodynamic properties of the system [4]. As a powerful computational tool, MD is an emerging computerized material technology, combines theoretical methods and computer technology to simulate molecular motion. It can simulate the physical mechanics and other properties of materials from the perspective of molecules. MD intuitively studies the morphological changes and molecular movement of materials at the molecular level. Compared with physical experiments and theoretical calculations, MDs have been a key tool for asphaltene research [5,6,7]. MD can analyze the diffusion process of regenerants in asphalt and their interaction with asphalt components at the molecular level. By simulating the movement paths and diffusion properties of regenerant molecules within the asphalt matrix, it is possible to deeply explore the mechanism of how regenerants act on the microstructure of asphalt. Gong et al. [8] studied the effects of warm-mix recycling on the thermodynamic properties of aged asphalt mixtures through MD. Cui et al. [9] utilized MD to investigate the mutual diffusion characteristics and crack resistance of virgin and aged asphalt binders. Yang et al. [10] analyzed the viscosity and self-healing properties of WMA-R by combining MD with laboratory tests. Furthermore, the effective diffusion of warm-mix regenerants is directly linked to the high-temperature rutting resistance and low-temperature cracking resistance of recycled asphalt mixtures. Therefore, establishing a mapping relationship between the diffusion properties of regenerants and the macroscopic performance of mixtures is of great significance. This study aims to systematically investigate the diffusion characteristics of biomimetic-based warm-mix regenerant in aged asphalt using MD, analyze the relationship between biomimetic-based warm-mix regenerant molecular structure and diffusion efficiency, and further explore the influence of diffusion behavior on the high- and low-temperature performance of asphalt mixtures. By combining experimental validation, a quantitative mapping model between biomimetic-based warm-mix regenerant diffusion properties and mixture performance will be established, providing a scientific basis for optimizing and applying warm-mix recycling technology. The findings will not only advance asphalt pavement recycling techniques but also offer theoretical support for the development of green transportation infrastructure

## 2. Test Materials and Optimal Dosage Determination

### 2.1. New Asphalt and Aggregate

SBS-modified asphalt was used in this experiment with the technical parameters listed in Table 1.

The test results in Table 1 show that all indicators of the SBS-modified asphalt used in this experiment meet the requirements of the specification.

The new aggregate is made of limestone and the new aggregate is divided into three levels: 0–5 mm, 5–10 mm, and 10–20 mm. The filler is made of finely ground limestone powder. The test results are shown in Table 2 and Table 3. The results all meet the requirements of the specification.

The test results in the table above indicate that the aggregates used meet all the technical requirements specified in the relevant standards.

### 2.2. RAP

The aged asphalt in this experiment was recycled and reused by RAP on the upper layer of the main road of a city. SBS-modified asphalt mixture AC-13 was used the upper layer. The upper layer material can be divided into three levels after screening, including 0–5 mm, 5–10 mm, and 10–15 mm. The three levels are coarse RAP, medium RAP, and fine RAP. The RAP screening results of milling materials are shown in Table 4.

According to Table 4, the content of coarse aggregate in RAP is relatively small. The reason may be that as the road life increases, the coarse aggregate plays a skeleton role in the asphalt mixture under the repeated action of driving loads, resulting in a decrease in the use performance of the road surface and weak resistance to deformation [13].

By burning RAP, the asphalt content in RAP was determined. The mass change ratio of each grade of RAP was used as each grade of material in RAP and the results are shown in Table 5.

### 2.3. Biomimetic-Based Warm-Mix Regenerant

**Figure 1:**
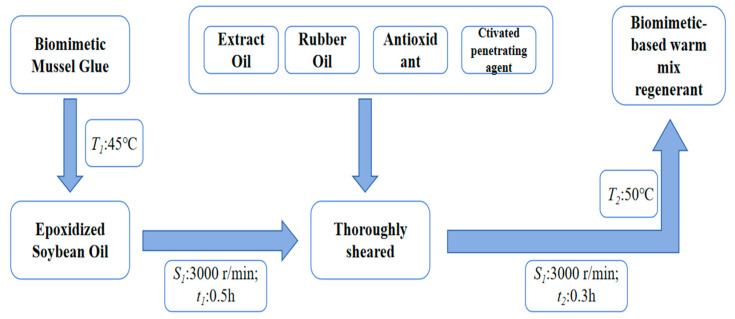
Flow chart of the preparation of biomimetic-based warm-mix regenerant.

This article uses homemade biomimetic-based warm-mix regenerant (the synthetic technology of novel bionic mussel glue is applied to warm mixing and recycling of recycled asphalt; a warm-mix regenerant with good warm-mixing and regenerating effects is developed based on the biocompatibility and high adhesion strength of bionic mussel glue). The biomimetic mussel glue was synthesized by Shanghai Aladdin Biochemical Technology Co., Ltd. (Shanghai, China), using the following reagent-grade raw materials: polytetramethylene ether glycol (MW = 2000 Da), 2,6-toluene diisocyanate (MW = 250.25 Da), and 1,4-butanediol (MW = 90.12 Da). The synthetic procedure is illustrated in Figure 1. Among them, the content of biomimetic-based mussel glue is A%, base oil is B%, extract oil is C%, active penetrant is D%, and antioxidant is E%. The sum of all parts from A to E is 100%. The performance indicators and test standards of warm-mixed regenerators are shown in Table 6.

The results in Table 4 show that the biomimetic-based warm-mix regenerant has a viscosity of 74 mm^2^/s at 60 °C, which falls within the appropriate range and facilitates mixing and compaction during construction. Its flash point reaches 237 °C, exceeding the standard, indicating high safety during storage, transportation and construction, good high-temperature stability, and low likelihood of accidental combustion or performance degradation. The contents of saturates and aromatics are appropriately proportioned to maintain the stability of the asphalt colloidal structure. After the thin-film oven test, the viscosity ratio and mass change meet the requirements, demonstrating excellent anti-aging performance and effectively extending the service life of asphalt pavements. The measured density is stable, ensuring uniform dispersion of the regenerant in asphalt. Collectively, these properties provide a solid foundation for the application of biomimetic-based warm-mix regenerant in asphalt recycling research, ensuring it can effectively restore and enhance the performance of aged asphalt, and offering a reliable material basis for asphalt recycling research.

### 2.4. Optimal Dosage Determination

**Figure 2:**
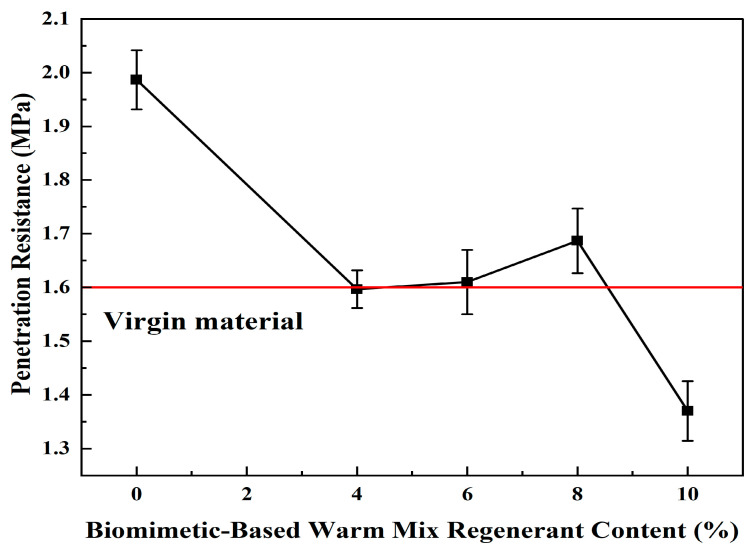

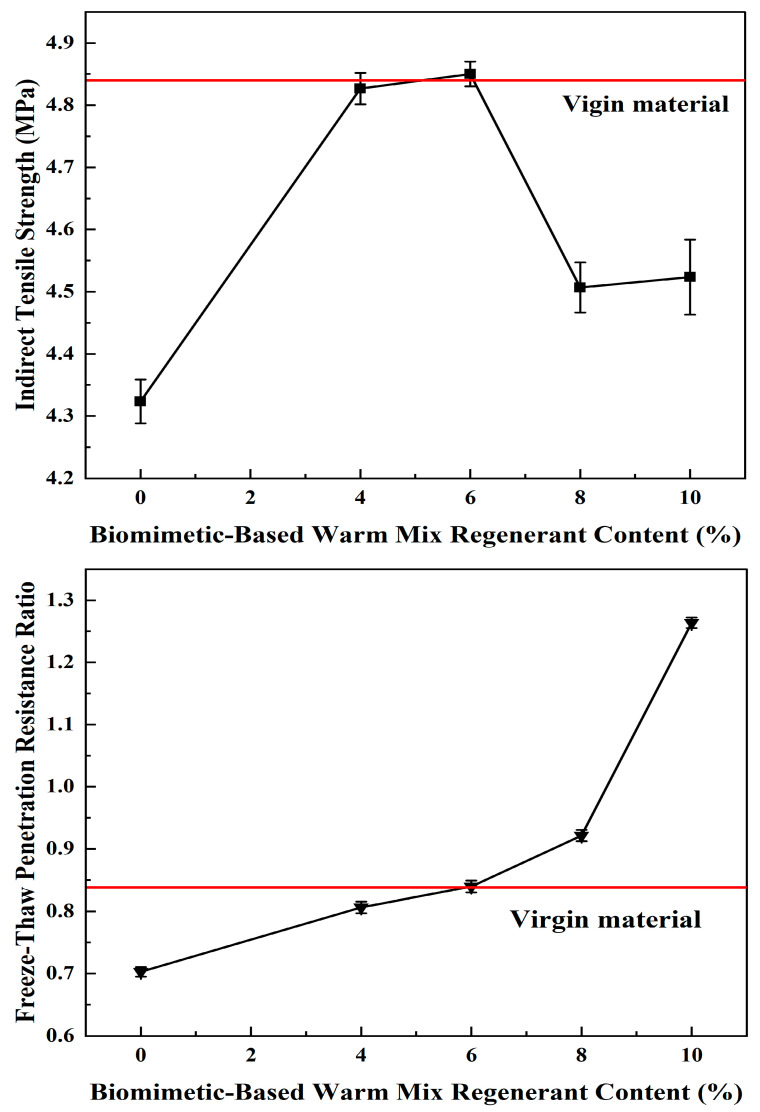
Experimental results.

Through uniaxial penetration, low-temperature splitting, and freeze-thaw penetration tests, the high-temperature, low-temperature, and water stability properties of the WMA-R mixture were determined, respectively, thereby determining the optimal amount of the best biomimetic-based warm-mix regenerant. The regenerator was added to RAP with 4%, 6%, 8%, and 10% biomimetic-based warm-mix regenerant of the old asphalt content. The test results are shown in Table 7 and Figure 2.

Through performance comparison tests between virgin materials and WMA-R mixtures with different regenerant dosages, the results show that when the biomimetic-based warm-mix regenerant dosage is 4%, the penetration strength of the mixture is comparable to that of virgin materials; when the dosage increases to 6%, its splitting strength is higher than that of virgin materials, the difference in penetration strength after freeze-thaw cycles is the smallest compared with virgin materials, and the conventional penetration strength is basically the same as that of virgin materials. Based on the comprehensive evaluation of penetration strength, splitting strength, and freeze-thaw performance, the RAP exhibits the best performance restoration effect at a 6% dosage. Therefore, 6% is selected as the baseline dosage for subsequent tests.

By comparing the performance of the new material with the mixture of WMA-R at different dosages, the recovery performance of aged asphalt at 6% dosage is better, so the subsequent test is performed with a dosage of 6%.

## 3. Test Content and Methods

### 3.1. MD

#### 3.1.1. Basic Principles of MD

MD is a computational simulation technique grounded in classical mechanics, designed to model many-body systems composed of numerous interacting particles. This method assumes that each particle within the system adheres to Newton’s laws of motion under the influence of a classical force field. By calculating the positions and interactions of particles, the specified initial conditions such as initial positions, velocities, and temperature are incorporated into the equations of Newtonian mechanics to determine the velocities and positional coordinates of particles at any given time point in the simulated system [14]. The fundamental principle underlying MD lies in the numerical integration of Newton’s equations of motion, which enables the simulation of molecular system dynamics. Newton’s second law is expressed as *F*(*X*) = *ma* = $\frac{dp}{dt}$, where *m* represents the particle’s mass, *a* is acceleration, *F* is the force acting on the particle, and *p* is the particle’s momentum. This paper investigates the mapping between the diffusion characteristics of WMA-R and the high-/low-temperature performance of mixtures based on MD.

There are many algorithms for solving Newton’s equations of motion, including the Verlet algorithm, velocity-Verlet algorithm, leap-frog algorithm, Gear predictor-corrector method, integration algorithms, and Beeman algorithm. Commonly used integration algorithms include:

1. Verlet algorithm: This offers high computational efficiency and achieves good accuracy with low computational cost, but performs less precisely in handling high-frequency vibrational modes.
2. Leap-frog algorithm: This second-order accuracy method is suitable for large-scale system simulations but shows weaker adaptability to complex systems compared to higher-order algorithms.
3. Velocity-Verlet algorithm: Known for its fast computation speed, high accuracy, and good stability, it is widely used in current applications.

**Figure 3:**
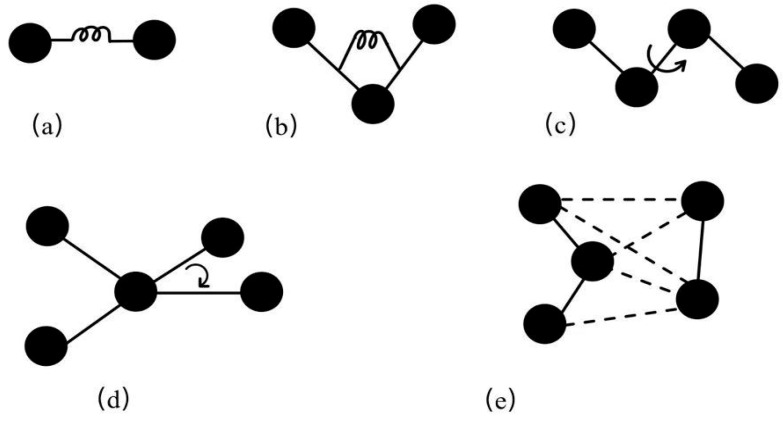
Energy terms. (**a**) Bond stretching, (**b**) angle bending, (**c**) dihedral angle, (**d**) improper torsion, (**e**) non-bonded [17].

A molecular force field is a mathematical model describing intermolecular interactions. Most molecular force fields describe the effects of various interaction forces on molecular potential energy through potential energy functions, including bonded and non-bonded interactions. Bonded interactions consist of bond stretching energy, angle bending energy, and dihedral torsion energy. Non-bonded interactions include van der Waals energy, electrostatic interaction energy, and hydrogen bonding energy [15]. These energy terms are illustrated in Figure 3. Force field parameters are typically derived from experiments and quantum mechanical calculations, to describe entire classes of molecules with reasonable accuracy [16].

The choice of force field is critical as it determines the strength and range of intermolecular interactions. Common force fields in simulation software include the COMPASS force field, COMPASS II force field, Dreiding force field, Universal force field, CVFF, PCFF, and GAFF, each applicable to different simulation environments. In this study, the aged SBS asphalt model was optimized under the COMPASS II force field, and the COMPASS force field was selected to geometrically optimize the constructed WMA-R molecules.

An ensemble is a collection of numerous identical, independent systems under specific macroscopic conditions, each existing in different microscopic states but exhibiting identical macroscopic properties. Common ensembles include Canonical ensemble (NVT), Microcanonical ensemble (NVE), Isothermal-Isobaric ensemble (NPT), Isobaric-Isoenthalpic ensemble (NPH), and Grand Canonical ensemble (µVT). Among these, the first three are most frequently employed. Based on this, the NVT ensemble and NPT ensemble were used to perform kinetic optimization on the molecular models.

**Figure 4:**
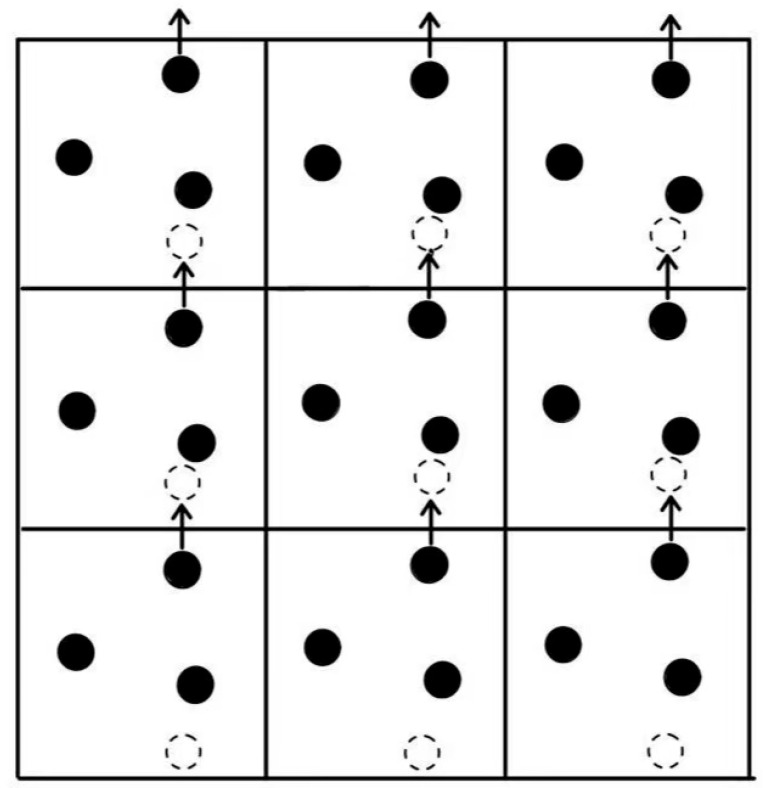
Periodic boundary conditions.

Molecular dynamics simulations face challenges handling massive particle quantities, where boundary conditions provide effective solutions. Boundary conditions mainly divide into periodic and non-periodic types. Periodic boundary conditions, one of the most prevalent approaches, are particularly suitable for simulating infinite systems. By treating the simulation domain as a repeating unit, particles reaching boundaries instantly reappear on the opposite side. This method effectively minimizes boundary effects in large-scale system simulations, with Figure 4 clearly illustrating its mechanism. Conversely, non-periodic boundary conditions prohibit particle re-entry once they exit the simulation domain.

In this study, MD was employed to establish molecular models of aged SBS asphalt and biomimetic-based warm-mix regenerant, aiming to investigate the diffusion characteristics of the warm-mix regenerant in aged asphalt and reveal the diffusion behavior of the regenerant in asphalt and its interactions with asphalt components at the molecular scale.

#### 3.1.2. Establishment of SBS Asphalt Molecules and the Biomimetic-Based Warm-Mix Regenerant Model

The theory of the colloidal structure of asphalt starts from the migration of chemical components. It is believed that after asphalt aging, the migration of components will lead to more of some components and less of other components. Inconsistent proportions between the components will lead to a reduced performance of asphalt roads. If the components can be adjusted by adding regenerators, the asphalt will restore its original properties. The compatibility theory of asphalt regeneration is based on chemical thermodynamics and believes that the compatibility of each component in the asphalt colloid system will decrease. The increase in the difference in solubility parameters between components will lead to the aging of asphalt. If a certain biomimetic-based warm-mix regenerant can be added to reduce its solubility parameters, the asphalt can recover or even exceed its original properties.

According to the above two theories, aging asphalt regeneration means better-dispersing asphaltene in aromatic and saturated parts under the action of colloids, forming a stable colloidal structure, and the rheological properties of asphalt being changed, so that the asphalt performance meets the requirements of quality indicators [18]. That is, mix the soft components rich in aromatic parts into aged bitumen in a certain proportion to create a new and more reasonable bitumen component. According to the asphalt colloid structure theory, based on the research on aging performance and grade classification, appropriate regeneration methods are selected for the aging degree of recovered asphalt. While improving the rheology performance of regenerated asphalt, the amount of cementitious materials on the old asphalt pavement should be increased as much as possible [19]. The method is based on component harmony and compatibility theory as the theoretical basis. The specific measures usually involve the addition of new asphalt materials and regenerators.

This study adopts an SBS-modified asphalt model and the experimental results of the four-component content are shown in Table 8.

**Figure 5:**
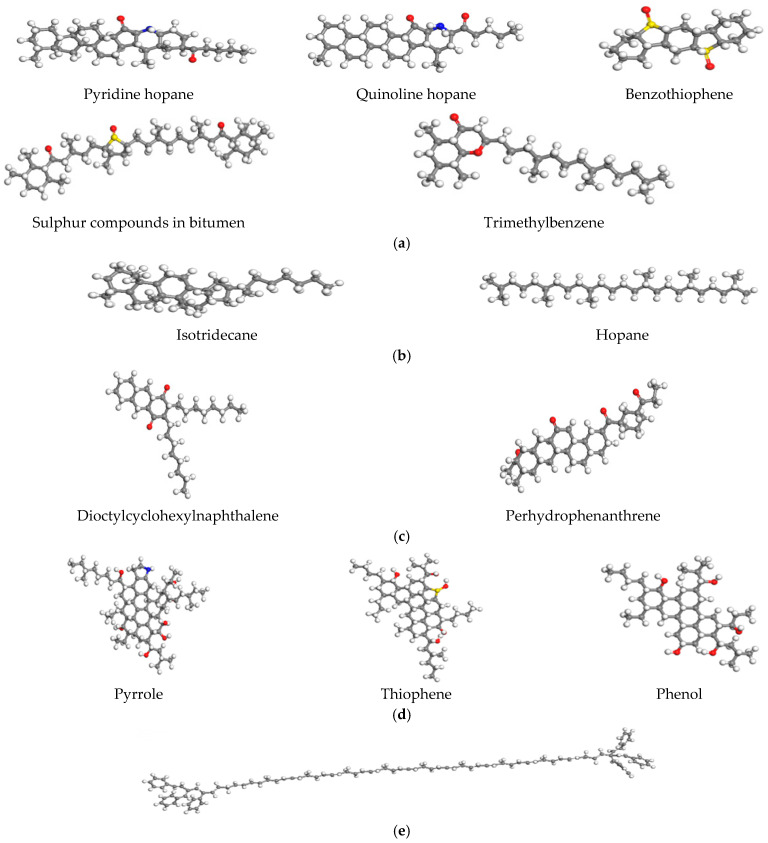
Structural model of the components of SBS-aged asphalt. (**a**) Colloid, (**b**) saturate fraction, (**c**) aromatic fraction, (**d**) asphaltene, (**e**) SBS.

To more accurately reflect the micro-properties of asphalt materials, the molecular structures of each component are shown in Figure 5. In the following figure, the red, blue, and yellow parts represent oxygen atoms, nitrogen atoms, and sulfur atoms, respectively. Due to the limitations of computer capabilities, this project will control the number of atoms in the matrix asphalt model. Therefore, it is impossible to directly convert equally based on the macro data of the four components of matrix asphalt during modeling, so this project will be adjusted by manual trial calculation. This project constructed the SBS-aged asphalt molecular model under the premise that the overall molecular mass of the control model is similar and the proportion of the four components obtained by manual calculation is similar to the actual experimental results.

**Figure 6:**
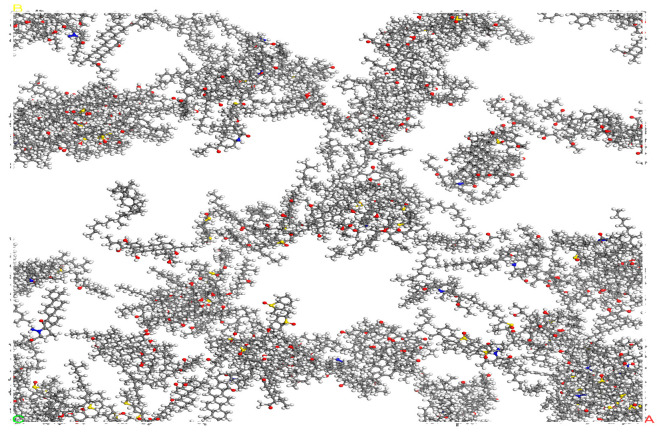
Model of SBS-aged asphalt.

To make the system model closer to the real material, the structure optimization is performed through Geometry Optimization in the Forcite module and the energy convergence is set to be sufficient. Then, under the Compass II force field, the structure is annealed 10 cycles to cross the energy barrier to find the global optimal low-energy conformation. Finally, under the NPT and NVT ensembles, the asphalt model is brought to an equilibrium state and the asphalt is relaxed and balanced as shown in Figure 6.

**Figure 7:**
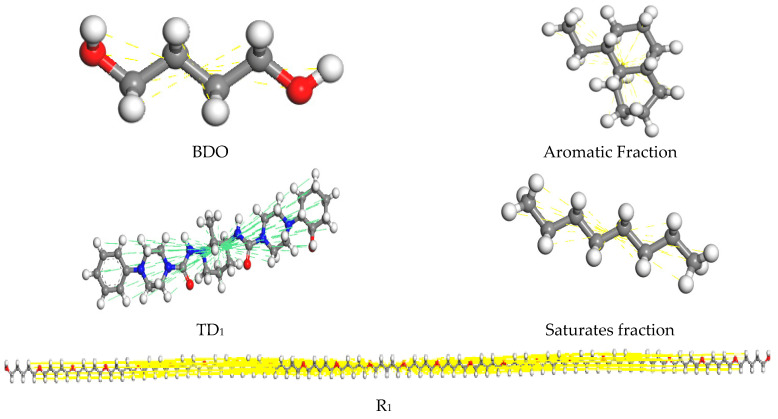
Model of biomimetic-based warm-mix regenerant.

The Amorphous Cell module of MS 2019 was used to construct a molecular model of the effect of biomimetic-based warm-mix regenerant with three-dimensional periodic boundary conditions, as shown in Figure 7. The specific operation is as follows: assemble a biomimetic-based warm-mix regenerant chain with asphalt molecules, calculate the mass fraction of the module, and determine the dosage of the biomimetic-based warm-mix regenerant to be 6%.

**Figure 8:**
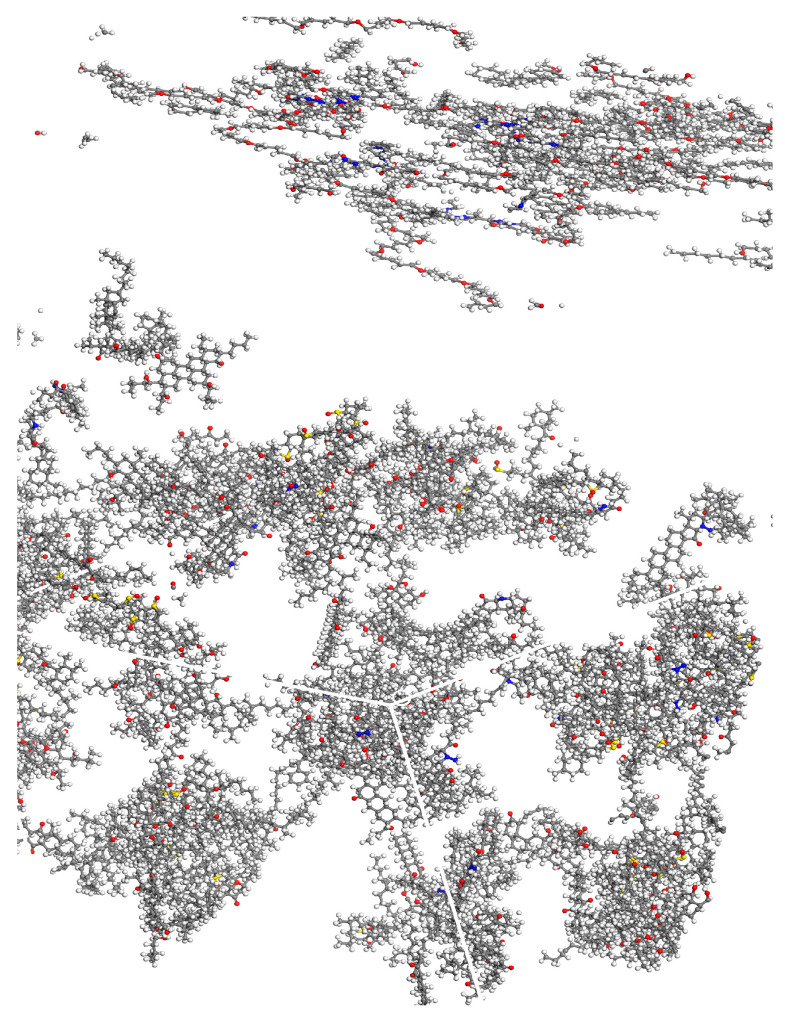
Initial state molecular model of the effect of biomimetic-based warm-mix regenerant.

**Figure 9:**
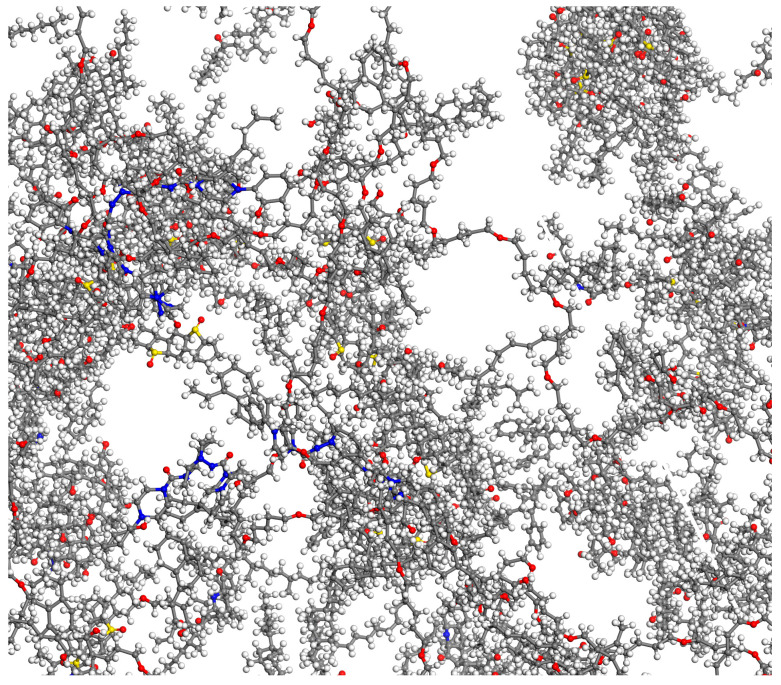
Molecular model of the fusion state of the effect of biomimetic-based warm-mix regenerant.

Finally, the molecular model of the biomimetic-based warm-mix regenerant is obtained as shown in Figure 8 and Figure 9. The upper part of Figure 8 represents Biomimetic-based warm-mix regenerant and the lower part represents Aged asphalt.

Then, geometric optimization was performed on the constructed WMA-R molecules, and the COMPASS force field was selected, with 100,000 iterations. Van der Waals forces were calculated by the atom-based method and the electrostatic interactions were calculated by the Ewald method. When the system energy drops and stabilizes, annealing is performed within the temperature range of 300–500 K and the number of iteration steps is set to 50,000 steps.

To make the molecular model stable in energy and volume, kinetic optimization of the molecular model that completes annealing is also required. The molecular dynamics optimization process is as follows: first, the constant temperature and constant volume (NVT) ensemble is selected for 200 ps dynamic calculations. This step is to simulate the diffusion in bitumen; then the constant temperature and constant pressure (NPT) ensemble is performed for 200 ps dynamic calculations; finally, the constant temperature and constant volume (NVT) ensemble is performed to gradually stabilize the system energy, and finally, a more stable WMA-R model is obtained. During the entire simulation process 298 K temperature is selected, Andersen is selected for the temperature controller, and Berendsen is selected for the pressure controller.

#### 3.1.3. Mean Square Displacement and the Diffusion Coefficient of Asphalt

Mean square displacement (MSD) refers to the average distance between the position of molecule I after moving over time relative to the reference position and can be used to evaluate the degree of diffusion of the molecule. The mean square displacement is linearly related to time. The larger the mean square displacement per unit of time, the faster the diffusion speed of the molecule, and the greater the diffusion coefficient of the molecule. Mean square displacement can characterize the average distance between molecules of different components of biomimetic-based warm-mix regenerant diffused in aged asphalt. Fitting the mean square displacement curve within a fixed period can be used to study the diffusion rate of different components of biomimetic-based warm-mix regenerant in aged asphalt [20].

In the Forcite module, the simulation process is carried out under the NPT ensemble of molecular quantity, pressure, and temperature constant [21], setting the pressure to atmospheric pressure (0.1 MPa), ensuring that the diffusion of the biomimetic-based warm-mix regenerant components in aged asphalt is carried out in the atmospheric environment. Set the simulation time of diffusion to 120 ps to ensure that the correlation coefficient of the mean square displacement fitting curve of different components of the biomimetic-based warm-mix regenerant is at a high level. The high- and low-temperature performance of the WMA-R was evaluated by a rutting test and low temperature bending test. Therefore, the simulated temperature was set to 263 K and 333 K. The mean square displacement curves of the biomimetic-based warm-mix regenerant components at these two temperatures were analyzed. Finally, the mean square displacement curves of the biomimetic-based warm-mix regenerant components were linearly fitted to obtain the diffusion coefficient of the biomimetic-based warm-mix regenerant in aged asphalt. Finally, the diffusion coefficient of the aged asphalt was compared with the diffusion coefficient of the biomimetic-based warm-mix regenerant in aged asphalt. Considering that asphalt aging is mainly based on aromatic and saturated diffusion rates, see Equation (1) to calculate the diffusion coefficient.

(1) $$\underset{t\to \infty }{\lim}\frac{1}{6t}*MSD(t)=\frac{1}{6}k$$

Among them, D is the diffusion coefficient, t is the time, and k is the slope of the mean square displacement and time. According to Formula (1), the diffusion coefficients of aged asphalt and regenerated asphalt were calculated at two temperatures: 263 K and 333 K.

### 3.2. Performance of Warm-Mixed Regenerated Asphalt Mixture

#### 3.2.1. Match Ratio Design

**Figure 10:**
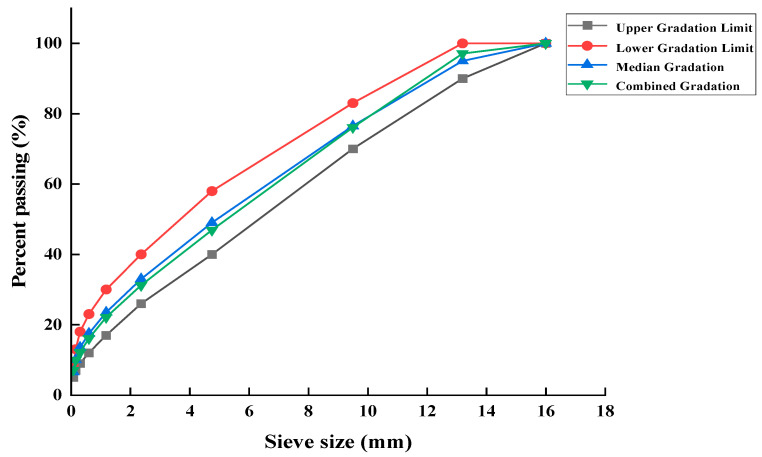
Synthetic gradation curve at 30% RAP content.

When conducting mix performance tests, the mix ratio between RAP and new aggregates must meet the requirements of the asphalt mixture grading design specifications. 30% RAP is used instead of new materials. The raw material grading and grading ranges are shown in Table 9 and the grading curve is shown in Figure 10. Meanwhile, the AC-13 gradation provides an appropriate skeletal structure with 40–60% passing rate at 4.75 mm sieve, which not only ensures sufficient contact between the regenerant and aged asphalt, but also maintains a dynamic stability exceeding 3000 cycles/mm under low-temperature mixing conditions while preserving excellent workability during construction. The AC-13 type grading median is used as the synthetic grading.

#### 3.2.2. Optimum Oil-Stone Ratio

According to the determined grading composition, five different oil and stone ratios with certain gradient differences were selected, including 4.6%, 4.7%, 4.8%, 4.9%, and 5.0%, and Marshall’s experiments were performed. First, heat the RAP material (135 °C) and new aggregate (160 °C); dry mix the RAP and new aggregate, then add asphalt and biomimetic-based warm-mix regenerant for wet-mixing to ensure uniformity, with the mixing temperature set at 130 °C; finally, fill the mixture into the test mold and compact it 50 times on each side. This operation is carried out according to the detailed standards and specification procedures stipulated in the current “Technical Specifications for Highway Asphalt Pavement Recycling” (JTG/T 5521-2019), and the optimal oil-stone ratio is finally determined. The test results are shown in Table 10.

**Figure 11:**
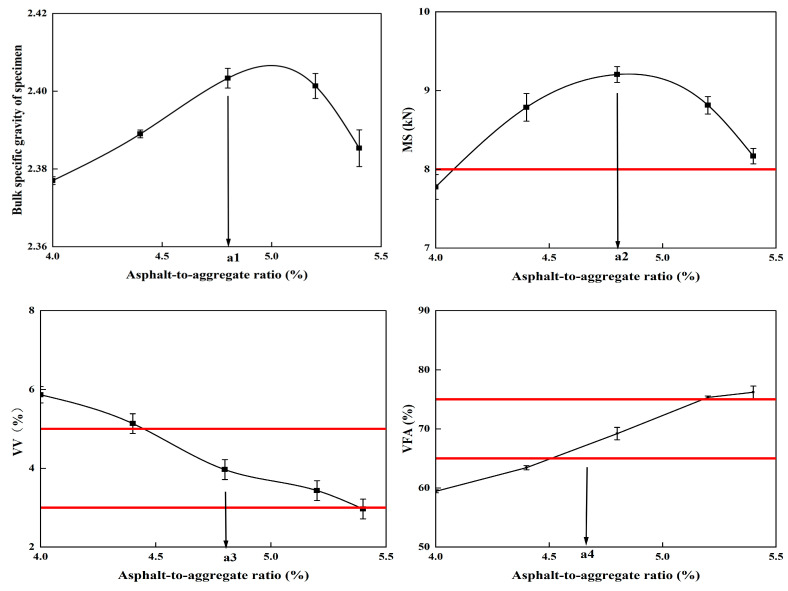

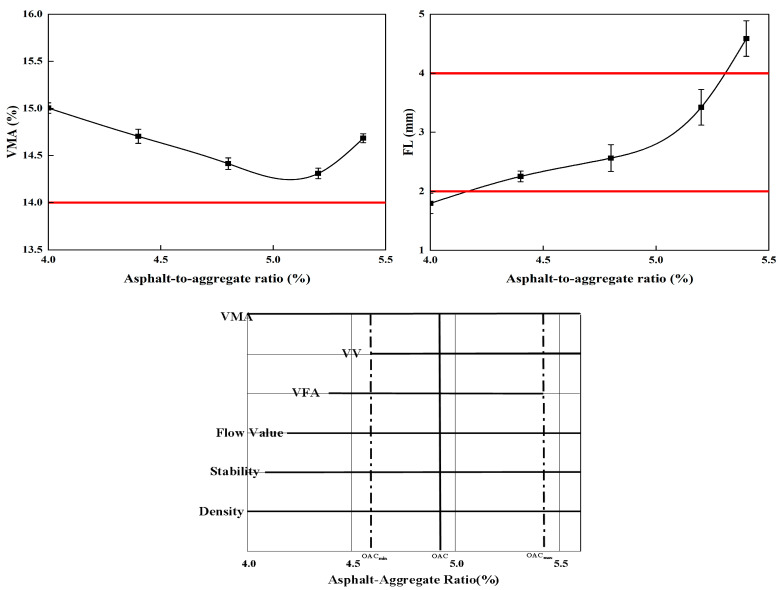
Graph of analyzed curve of the Marshall test.

According to the Marshall test results of Table 10, the requirements of the “Technical Specifications for Highway Asphalt Pavement Recycling” (JTG/T 5521-2019) and the “Technical Specifications for Construction of Highway Asphalt Pavements” (JTG F40-2004), the relationship curve charts are drawn with the oil-stone ratio as the horizontal coordinate and the Marshall test indicator as the vertical coordinate, as shown in Figure 11.

Through analysis and calculation, the initial value of the best oil-stone ratio OAC1 = 4.78%, OAC2 = 4.8%, and the final determination of the best oil-stone ratio OAC is (OAC1 + OAC2)/2 = 4.8%. The design mix ratio is inspected and all indicators meet the specification requirements.

#### 3.2.3. Rutting Test

Generally speaking, the high-temperature performance of asphalt mixture refers to the ability of asphalt pavement to resist vehicle deformation under high-temperature environments [24]. Higher temperatures have a certain impact on the asphalt mixture, which can easily lead to asphalt aging or accelerate its aging. During use, the asphalt mixture is prone to irrecoverable deformation when passing through vehicle load and environmental impact at higher temperatures, and usually, but is the most common form of damage. The rut rolling depth of the rut tester is used to calculate the dynamic stability of the regenerated mixture. At 60 °C, the test piece was repeatedly rolled with a wheel load of 70 MPa at a speed of 42 times/min ± 1 times/min. Dynamic stability is used to evaluate the high-temperature rut-resistant deformation ability of the asphalt mixture; see Equation (2).

(2) $$\mathrm{DS}=\frac{(t_{2}-t_{1})\times N}{d_{2}-d_{1}}\times C_{1}\times C_{2}$$

where DS is the dynamic stability of the asphalt mixture (times/mm), t_1_ = 45 min, t_2_ = 60 min, d_1_ is the deformation of the specimen at 45 min, and d_2_ is the deformation of the specimen at 60 min.

#### 3.2.4. Low-Temperature Performance

Under the action of driving load under the asphalt pavement in a low-temperature environment, the pavement is prone to damage due to breakage. According to domestic and foreign research, the low-temperature performance of asphalt mixture is one of the main properties of asphalt pavement, and its importance is very important compared to other properties [25,26,27,28,29]. When the asphalt pavement is in a low-temperature environment, the temperature of the asphalt pavement will also become lower, resulting in asphalt pavement shrinkage. When the stress generated by the shrinkage of the mixture is greater than the maximum bending tensile strength that the asphalt mixture itself can bear, the material inside the asphalt pavement will be broken, resulting in cracks. After cracks are generated, natural water and groundwater are immersed, resulting in more serious damage to the asphalt pavement. The produced beam specimens were stored at low temperature for 4 h at −10 °C, and loaded at a loading speed of 50 min/min and 40 KN. For the low-temperature beam bending failure test, the calculation formula is shown in Equation (3).

(3) $$\varepsilon_{B}=\frac{6\times h\times d}{L^{2}}$$

where *h* is the maximum tensile strain of the specimen, which is calculated as the cross-span height (mm) divided by a certain value, *d* is the mid-span deflection (mm), and *L* is the span of the specimen (mm).

### 3.3. Research on Energy Consumption and Carbon Emission of Warm-Mix Recycled Asphalt Mixture

Conventional hot-mix asphalt mixtures generate a series of toxic gases during mixing, paving, and compaction, causing significant impacts on the natural environment. In this project, the carbon emission factor method (using unit carbon emissions as the measurement indicator) was adopted. Based on the carbon emission factors provided by the Intergovernmental Panel on Climate Change (IPPC) and using the Life Cycle Analysis (LCA) method, the construction of asphalt pavements was divided into four stages: raw material production, raw material transportation, mixture production, and mixture construction. By establishing a carbon emission model during asphalt construction, quantitative analyses were conducted on the carbon emissions of operations in each stage.

For calculation convenience and unified measurement indicators, it was finally determined that the carbon emissions generated from the production and use of 1 ton of asphalt mixture would serve as the carbon emission measurement indicator (CMI) for each link in all stages, with the total carbon emissions from the production and use of 1 ton of asphalt mixture as the overall CMI for asphalt mixtures.

The raw material production for WMA-R pavements includes the production of recycled old materials (RAP), new asphalt, and new aggregates. An evaluation model for the production stage was established to obtain the total carbon emissions and energy consumption for this stage. The calculation of old material production is shown in Equation (4).

*M*_1_ = (*H*_1_/*W*_0_) × *P* (4)

In the formula, *M*_1_, *H*_1_, *P*, and *W*_0_ represent the emission amount of a certain greenhouse gas (kg), fuel consumption per operating hour of the milling machine (L/h), carbon emission factor of unit volume diesel, and the amount of old material produced by the milling machine per hour (t/h).

The model for the raw material transportation stage adopts the transportation distance method and the calculation is shown in Equation (5):

*M*_2_ = *S*_1_ × *L* × *P* (5)

In the formula, *M*_2_, *S*_1_, *L*, and *P* denote the emission amount of greenhouse gases (MJ), fuel consumption per kilometer during comprehensive vehicle transportation (kg), average transportation distance of the vehicle (km), and carbon emission factory.

Considering the application of thermodynamics principles and based on the laws of heat transfer and energy conservation, the fuel consumption during the mixing process of asphalt mixtures is calculated as shown in Equations (6) and (7):

(6) $$m_{ac}=\frac{c_{a}m_{a}\Delta t_{a}}{\eta_{1}\lambda_{1}}$$

For Equation (6) *m_ac_*, *m_a_*, *c_a_*, Δ*t_a_*, *η*_1_, and *λ*_1_ represent the diesel consumption for asphalt production (kg), mass of asphalt (kg), specific heat capacity of asphalt (MJ/(kg·°C)), heating temperature increment of asphalt (°C), efficiency of diesel combustion energy transferred to asphalt (%), and energy consumption coefficient of diesel (MJ/kg).

(7) $$m_{gz}=\frac{c_{g}m_{g}∆t_{g}+c_{w}m_{w}∆t_{w}}{\eta_{2}\eta_{3}\lambda_{2}}$$

For Equation (7) *m_gz_*, *m_g_*, *m_w_*, *c_g_*, *c_w_*, Δ*t_g_*, Δ*t_w_*, *λ*_2_, *η*_2_, and *η*_3_ represent heavy oil consumption for aggregate production (kg), mass of aggregate (t), mass of water (t), specific heat capacity of aggregate (MJ/(kg·°C)), specific heat capacity of water (MJ/(kg·°C)), heating temperature increment of aggregate (°C), heating temperature increment of water (°C), energy consumption coefficient of heavy oil (MJ/kg), heavy oil combustion efficiency (%), and drum heat exchange efficiency (%).

The gas emission amount M32 from material heating is obtained by multiplying the fuel consumption for heating asphalt and aggregates calculated by Equation (8) with the carbon emission factor, as shown in Equation (8):

*M*_32_ = *m* × *P* (8)

In the formula, *M*_32_, *m*, and *P* represent the emission amount of a certain greenhouse gas (kg), fuel consumed for heating asphalt and aggregates (kg), and the emission factor of a certain greenhouse gas per unit fuel.

The total emission amount of a certain greenhouse gas in the mixing stage is shown in Equation (9).

*M*_3_ = *M*_31_ + *M*_32_ + *M*_33_ (9)

The mixture construction stage mainly includes the paving and compaction stages, with the main emission sources being energy consumption of mechanical equipment and high-temperature emissions. The calculation is shown in Equation (10):

*M*_41_ = (*S*_4_/*W*_4_) × *P* (10)

In the formula, *M*_41_, *S*_4_, *W*_4_, and *P* represent the emission amount of a certain greenhouse gas (kg), fuel consumption per operating hour of the paver (L/h), amount of mixture paved per hour by the paver in practice (t/h), and carbon emission factor.

## 4. Experimental Results and Discussion

### 4.1. Analysis of Molecular Characteristics of Biomimetic-Based Warm-Mix Regenerant

**Figure 12:**
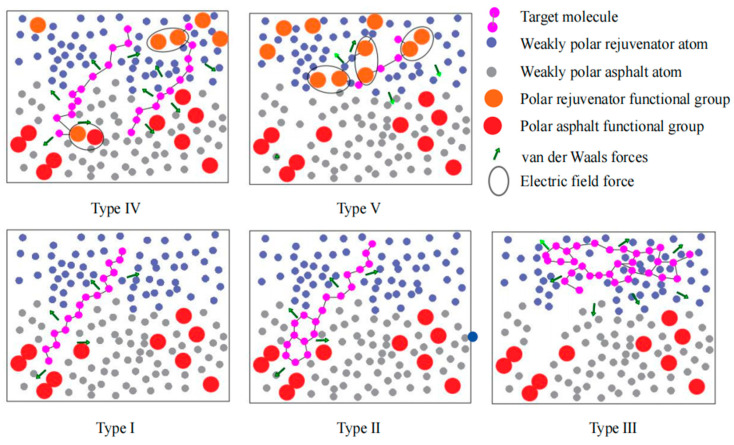
Molecular properties of biomimetic-based warm-mix regenerant.

The molecular characteristics analysis diagram of the biomimetic-based warm-mix regenerant is shown in Figure 12.

The five types of molecules are: (a) Type I: the typical molecule is saturated A; (b) Type II: the typical molecule is aromatic A and aromatic E; (c) Type III: the typical molecule is saturated B; (d) Type IV: the typical molecule is oleic acid and linoleic acid; (e) Type V: the typical molecule is glycerol.

**Figure 13:**
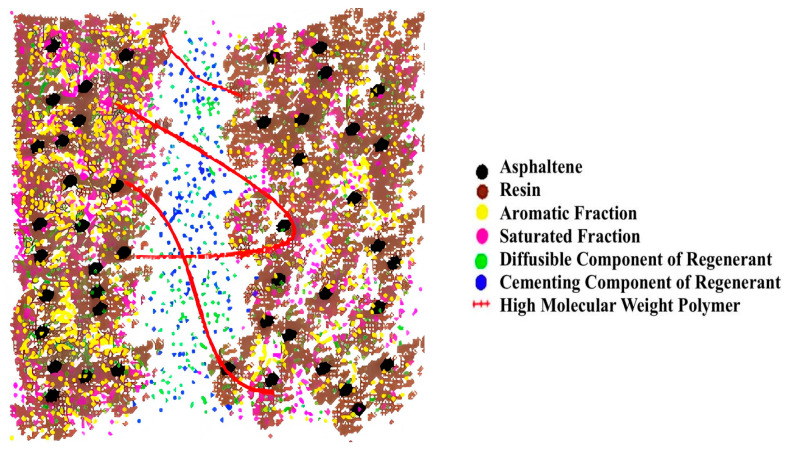
Schematic diagram of the mechanism of action of biomimetic-based warm-mix regenerant.

Figure 12 shows the molecular properties of the biomimetic-based warm-mix regenerant. It can be seen from the MS results that the biomimetic-based warm-mix regenerant molecules should be chain-like, low-polar, and have small cohesive energy, which is conducive to reducing viscosity, rapidly diffusing into the asphalt, avoiding volatility, and reducing costs. A schematic diagram of the mechanism of action of a biomimetic-based warm-mix regenerant is shown in Figure 13.

The auxiliary functional design of a biomimetic-based warm-mix regenerant includes cementitious component design (diluting the regenerator to promote uniform dispersion while reducing the mixing temperature) and interface enhancement design (using long-chain macromolecular polymers to enhance the interface, thereby improving interface performance). It has the following characteristics: 1. the activation and permeability components have various characteristics such as reducing viscosity and lowering the temperature; 2. the high molecular polymer can increase polarity and enhance interface performance; and 3. the cementitious components can restore the characteristics of aged asphalt.

### 4.2. Analysis of Diffusion Coefficient

The recovery of aged asphalt is analyzed by using the diffusion coefficient of the biomimetic-based warm-mix regenerant at temperatures of 263 K and 333 K. The diffusion coefficients are shown in Table 11.

Under 263 K conditions, with the addition of the biomimetic-based warm-mix regenerant, the diffusion coefficients of aromatic and saturated components increased significantly. The increase in the diffusion coefficient leads to a decrease in the viscosity of the regenerator and asphalt, thereby promoting mutual diffusion within the system, and restoring the low-temperature performance of aged asphalt to a certain extent. Similarly, under the high temperature of 333 K, the diffusion coefficients of aromatic and saturated components are also increased due to the addition of a biomimetic-based warm-mix regenerant, and the viscosity is reduced, which indicates that the high-temperature performance of asphalt has improved.

### 4.3. Analysis of Diffusion Coefficient and Mixing Performance Mapping

The high-temperature performance of the mixture can be characterized by rutting tests and the results of rutting tests are shown in Table 12.

According to the results of Table 12, with the addition of the biomimetic-based warm-mix regenerant, the dynamic stability is greatly improved, which can improve the high-temperature performance of the asphalt mixture and reduce the mixing and paving temperature. The diffusion coefficient of regenerated asphalt at 333 K temperature is improved compared with aged asphalt and is the same as the test conditions for the rut test, that is, the biomimetic-based warm-mix regenerant under molecular dynamics simulation has recovered the aged asphalt, and the performance of the mixture also proves each other.

The low-temperature performance of the mixture is characterized by a low-temperature beam bending test and the test results are shown in Table 13.

The beam specimens made of a mixture with a biomimetic-based warm-mix regenerant was subjected to a low-temperature beam bending test. The test results showed that the biomimetic-based warm-mix regenerant could effectively restore the low-temperature performance of aged SBS-modified asphalt mixture. The low-temperature stability of the warm-mixed asphalt mixture meets the specification requirements of ≥3000 and the low-temperature performance is good. In molecular dynamics simulation, the diffusion coefficient of regenerated asphalt was significantly improved under the temperature of 263 K, which was verified with the performance conclusions of the mixture.

### 4.4. Analysis of Carbon Emission and Economic Benefits

A systematic comparison of the energy consumption and carbon emissions between the biomimetic WMA-R mixture and the conventional hot-mix mixture is shown in Table 14.

The results indicate that the WMA-R mixture achieves 39.32% energy savings and 39.07% CO_2_ emission reduction compared to the conventional hot-mix asphalt mixture of the same type. This demonstrates that the addition of the biomimetic warm-mix regenerant significantly reduces carbon emissions.

The invention and application of the biomimetic warm-mix regenerant endow the WMA-R mixture with economic advantages over conventional hot-mix asphalt mixtures primarily in two aspects:

(1) During construction, hot-mix asphalt mixtures require high mixing temperatures (typically controlled at approximately 170 °C) to compensate for heat loss during transportation and paving, which consumes substantial energy. This study reveals that the use of the biomimetic warm-mix regenerant can reduce the mixing temperature while achieving the same performance as hot-mix asphalt mixtures, thereby minimizing energy consumption.
(2) The warm-mix recycling technology adopted in this study incorporates 30% RAP material, enabling its secondary utilization. On the one hand, this addresses the problem of land waste caused by RAP stockpiling; on the other hand, the recycling technology rejuvenates the aged asphalt in RAP, reducing the dosage of new asphalt and aggregates required for mixture preparation compared to hot-mix asphalt mixtures, thus saving raw material costs.

## 5. Conclusions

1. Through MD, we established a multi-scale computational model at the molecular level, significantly reducing experimental workload and shortening research cycles compared to conventional experimental methods. Within the temperature domain of 263 K (−10 °C) to 333 K (60 °C), the biomimetic-based warm-mix regenerant was demonstrated to enhance the diffusion coefficients of aromatic and saturate fractions in the asphalt system. By enhancing molecular-level interdiffusion between the regenerant and aged asphalt components, the targeted restoration of both high- and low-temperature performance was achieved. The establishment of a quantitative mapping model correlating “molecular diffusion coefficients with macroscopic performance parameters” provides atomic-level theoretical foundations for precision engineering of warm-mix regeneration technology, offering an innovative methodology for precision engineering of warm-mix regeneration technology, offering an innovative methodology for the intelligent design of sustainable pavement materials.
2. The biomimetic-based warm-mix rejuvenated asphalt mixture exhibited substantial improvement in dynamic stability compared to the aged control group. Particularly under 333 K high-temperature conditions, the enhancement in diffusion coefficient showed a significant positive correlation with dynamic stability gain, revealing the decisive role of molecular diffusion efficiency in high-temperature viscoelastic recovery. At 263 K conditions, the regenerant effectively restored the low-temperature performance of aged SBS-modified asphalt mixtures, demonstrating its molecular permeation mechanism in enhancing microscopic stress relaxation capacity. The developed three-tier association model of “regenerant molecular structure-diffusion kinetics parameters-mixture mechanical properties” establishes a traceable theoretical pathway for the molecular design of regenerants.
3. The biomimetic-based warm-mix regeneration technology achieves a 39.32% reduction in energy consumption and a 39.07% decrease in CO_2_ equivalent emissions compared to conventional hot-mix processes, overcoming the inherent “high-carbon remediation” paradox of traditional regeneration methods. This technology not only delivers immediate economic benefits through construction cost reduction but more importantly provides a key solution for the transportation sector to achieve global carbon neutrality goals through its low-carbon material system. Its large-scale application will catalyze the transformation of the asphalt industry towards a novel development paradigm featuring “molecular precision design-low-carbon process integration-full life-cycle management”, aligning with the coordinated development demands of resource recycling and ecological conservation under the “Dual Carbon” strategy, thereby establishing a replicable technological paradigm for high-quality economic growth and ecological civilization construction.

This study systematically investigated the diffusion characteristics of biomimetic-based warm-mix regenerant in aged asphalt and their effects on the high-/low-temperature performance of asphalt mixtures. Future research will expand the temperature range investigation to validate performance across broader thermal conditions, incorporate multi-dosage experiments and diverse mixture types to enhance conclusion universality, optimize regenerant formulations based on simulation results, and establish long-term performance monitoring mechanisms.

## Figures and Tables

**Table 1:** Main technical indicators of SBS-modified asphalt.

Evaluation Metrics	Test Results	Metric Requirements	Reference Standard [11]
Penetration at 25 °C (0.1 mm)	66	60~80	JTG E20: T0604
Softening Point (°C)	56	≥55	JTG E20: T0606
Ductility at 5 °C (cm)	37	≥30	JTG E20: T0605
Elastic Recovery (%)	72.7	≥65	JTG E20: T0662
Adhesion Grade	5	≥4	JTG E20: T0616

**Table 2:** Fine aggregate test results.

Test Items	Test Results	Metric Requirements	Reference Standard [12]
	0–5 mm		
Apparent relative density	2.733	≥2.45	JTG E42: T0328
Sand equivalent (%)	67	≥50	JTG E42: T0334

**Table 3:** Coarse aggregate test results.

Test Items	Test Results		Metric Requirements	Reference Standard [12]
	5–10 mm	10–20 mm		
Apparent relative density	2.745	2.697	≥2.45	JTG E42: T0304
Water absorption rate (%)	0.52	0.42	≤3.0	JTG E42: T0307
Content of flaky and elongated particles (%)	—	9.2	≤20	JTG E42: T0312
Content of particles <0.075 mm (%)	0.8	0.3	≤1.0	JTG E42: T0310

**Table 4:** RAP screening results of milling materials.

Sieve Size (mm)		16.0	13.2	9.5	4.75	2.36	1.18	0.6	0.3	0.15	0.075
Percent passing	Coarse RAP	100	96.1	52.4	16.2	10.6	7.6	4.8	1.4	0.5	0.2
	Medium RAP	100	100	99.8	61.8	40.7	28.7	16.9	7.7	3.1	1.2
	Fine RAP	100	100	100	89.8	57.5	37.6	19.5	7.7	2.9	1.1

**Table 5:** RAP asphalt content results.

Test Results			
Experimental Parameters	0–5 mm	5–10 mm	10–20 mm
Asphalt Content (%)	7.71	6.68	3.90

**Table 6:** Performance indexes of biomimetic-based warm-mix regenerant.

Item No.	Test Item	Test Result	Specification Requirement	Reference Standard [11]
1	Vscosity at 60 °C (mm^2^/s)	74	50–175	JTG E20: T0619
2	Flash point (°C)	237	≥220	JTG E20: T0611
3	Saturated component content (%)	16.6	≤30	JTG E20: T0618
4	Aromatic component content (%)	60.8	Field-Measured	JTG E20: T0618
5	Viscosity ratio before and after thin film oven test	1.018	≤3	JTG E20: T0619
6	Mass change after thin film oven test (%)	−1.24	≤4 ≥ −4	JTG E20: T0609 or JTG E20: T0610
7	Density at 15 °C (g/cm^3^)	1.001	Field-Measured	JTG E20: T0603

**Table 7:** Experimental results.

Biomimetic-Based Warm-Mix Regenerant Content (%)	Penetration Resistance (MPa)	Indirect Tensile Strength (MPa)	Freeze-Thaw Penetration Resistance Ratio
0	1.99	4.32	0.703
4	1.60	4.83	0.809
6	1.61	4.85	0.839
8	1.68	4.50	0.922
10	1.38	4.53	1.264
Virgin Aggregate	1.60	4.84	0.838

**Table 8:** The four-component content of asphalt.

Component Name	Component Content (%)
Aromatic fraction	31.48
Asphaltene	4.93
Resin	40.53
Saturated fraction	23.06

**Table 9:** Raw material grading and grading range.

Raw Materials	Percent Passing Each Sieve (%)									
	16	13.2	9.5	4.75	2.36	1.18	0.6	0.3	0.15	0.075
10–15	99	93.8	18.5	0.3	0.3	0.3	0.3	0.3	0.1	0.1
5–10	100	99.9	95.7	11.2	2.1	1.3	1.1	1.0	0.8	0.6
0–5	100	100	100	98.3	75.5	52.4	29.6	15.5	6.8	2.9
Mineral filler	100	100	100	100	100	100	100	100	100	100
RAP 0–5	100	96.1	52.4	16.2	10.6	7.6	4.8	1.4	0.5	0.2
RAP 5–10	100	100	99.8	61.8	40.7	28.7	16.9	7.7	3.1	1.2
RAP 10–20	100	100	100	89.8	57.5	37.6	19.5	7.7	2.9	1.1
Gradation upper limit	100	90	70	40	26	17	12	9	7	5
Gradation lower limit	100	100	83	58	40	30	23	18	13	8
Gradation median	100	95	76.5	49	33	23.5	17.5	13.5	10	6.5

**Table 10:** Marshall test results.

Asphalt-to-Aggregate Ratio (%)	4.0	4.4	4.8	5.2	5.4	Specification Requirements [22,23]
Bulk specific gravity of specimen	2.377	2.389	2.403	2.400	2.387	—
Theoretical maximum specific gravity	2.531	2.525	2.515	2.488	2.472	—
Air voids (VV) (%)	5.8	5.1	4.0	3.4	3.0	3~5
Voids in mineral aggregate (VMA) (%)	15	14.7	14.4	14.3	14.7	≥14
Voids filled with asphalt (VFA) (%)	59.4	63.4	69.1	75.3	76.6	65~75
Marshall stability (MS) (kN)	7.83	8.81	9.21	8.82	8.19	≥8
Flow value (FL) (mm)	1.87	2.26	2.54	3.47	4.56	2–4

**Table 11:** Diffusion coefficient.

Temperature (K)	Aged Aromatic Fraction	Aged Saturated Fraction	Rejuvenated Aromatic Fraction	Rejuvenated Saturated Fraction
263	6.6 × 10^−9^	1.16 × 10^−8^	4.0 × 10^−8^	3.83 × 10^−8^
333	8.0 × 10^−8^	6.0 × 10^−8^	8.6 × 10^−8^	8.5 × 10^−8^

**Table 12:** Rutting test results.

Warm-Mix Recycled Asphalt Mixture	DS (Cycles/mm)	Average Value	Specification Requirements	Test Method [11]
0% Additive Content	3210	2860	≥2400	JTG E20:T0719
	2510			
6% Additive Content	3231	4631		
	6462			
Virgin Material	5836	6233		
	6631			

**Table 13:** Low-temperature beam bending test.

Warm-Mix Recycled Asphalt Mixture	Maximum Flexural Tensile Strain	Average Value	Specification Requirements	Test Method [11]
0% Additive Content	2284	2370	≥3000	JTG E20:T0715
	2456			
6% Additive Content	3382	3432		
	3482			
Virgin Material	3182	3163		
	3144			

**Table 14:** Energy consumption and carbon emission of mixtures.

	Energy Consumption MJ		Carbon Emission kg	
	Warm-Mix Recycled Mixture	Conventional Mixture	Warm-Mix Recycled Mixture	Conventional Mixture
Production Stage	162.504	296.163	14.188	25.787
Transportation Stage	30.995	30.538	3.213	3.165
Mixing Stage	242.527	401.382	19.931	33.29
Construction Stage	14.675	14.675	1.523	1.523
Total	450.701	739.758	38.855	63.765

## Data Availability

The original contributions presented in this study are included in the article. Further inquiries can be directed to the corresponding author.
